# Supplementary material for: The causal role of circulating inflammatory markers in osteoporosis: a bidirectional Mendelian randomized study
Source: Front Immunol. 2024 Jul 18;15:1412298. doi: 10.3389/fimmu.2024.1412298 (PMC11291241; doi:10.3389/fimmu.2024.1412298)
Supplement: Supplementary file 1 [file DataSheet_1.zip › supplementary material/CIM-OSTEOPOROSIS /GCST90274787.csv_forest.pdf]

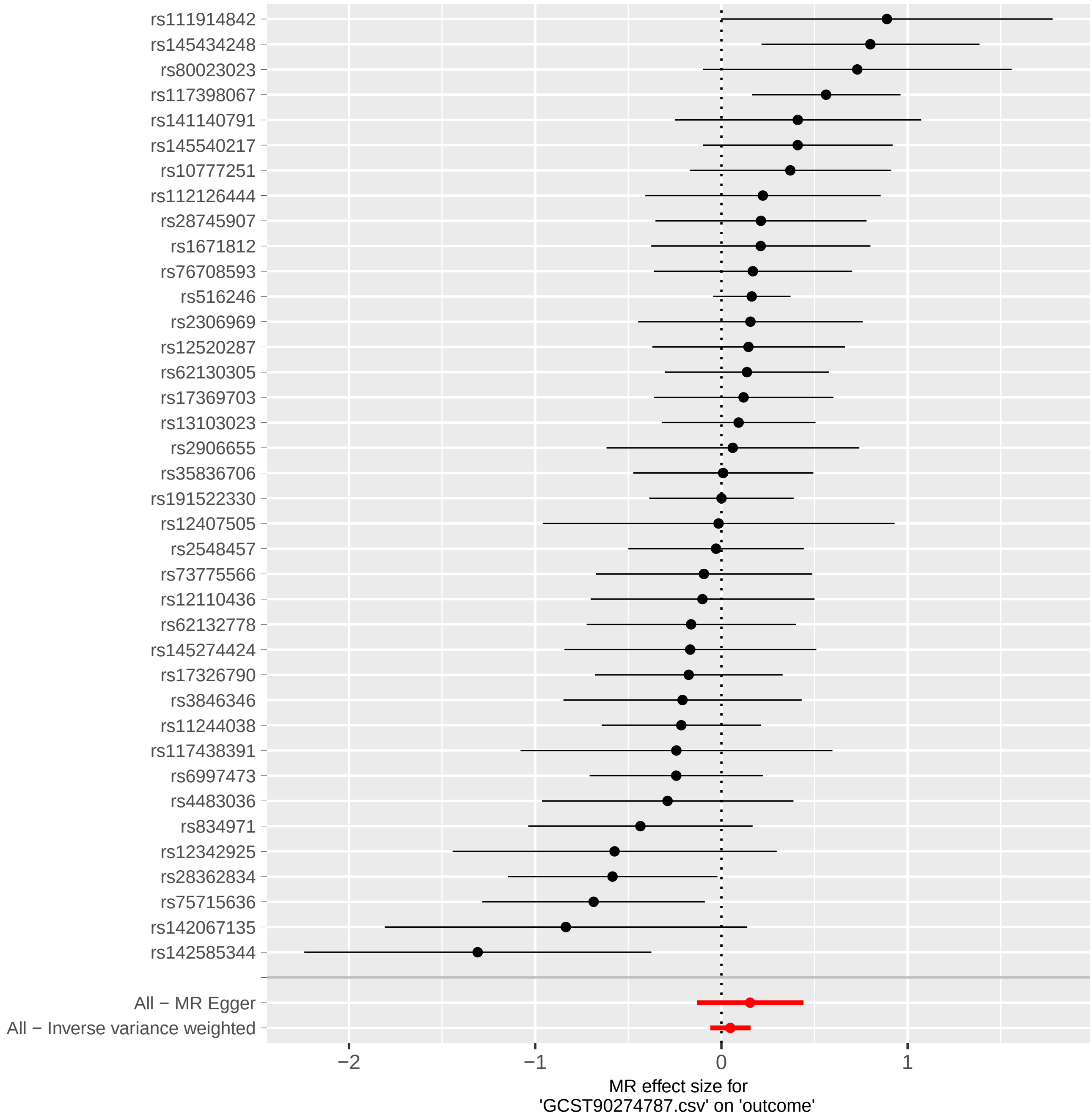
rs111914842
rs145434248
rs80023023
rs117398067
rs141140791
rs145540217
rs10777251
rs112126444
rs28745907
rs1671812
rs76708593
rs516246
rs2306969
rs12520287
rs62130305
rs17369703
rs13103023
rs2906655
rs35836706
rs191522330
rs12407505
rs2548457
rs73775566
rs12110436
rs62132778
rs145274424
rs17326790
rs3846346
rs11244038
rs117438391
rs6997473
rs4483036
rs834971
rs12342925
rs28362834
rs75715636
rs142067135
rs142585344
All − MR Egger
All − Inverse variance weighted
−2
−1
0
1
MR effect size for
'GCST90274787.csv' on 'outcome'
